# Low Birth Weight and Its Associated Factors among Newborns Delivered at Wolaita Sodo University Teaching and Referral Hospital, Southern Ethiopia, 2018

**DOI:** 10.1155/2019/4628301

**Published:** 2019-07-22

**Authors:** Eyasu Alem Lake, Robera Olana Fite

**Affiliations:** Department of Nursing, College of Health Science and Medicine, Wolaita Sodo University, Wolaita Sodo, Ethiopia

## Abstract

**Background:**

Birth weight has a vital role in determining newborns survival in vulnerable conditions. Low birth weight is associated with fetal and neonatal morbidity and mortality, impairment of growth and development and also chronic disease later in life. This study was aimed to assess the magnitude of low birth weight and its determinants in Wolaita Sodo University teaching and referral hospital, southern Ethiopia.

**Methods:**

Institution based cross-sectional study was conducted from November to December 2018. Systematic random sampling technique was used to select study participants. Data was collected by interviewing mothers through structured questionnaire and reviewing neonates' medical records using a checklist. Multivariable binary logistic regression analyses were employed to identify factors associated with neonatal jaundice.

**Results:**

The proportion of low birth weight in the study area was found to be 15.8% (95% CI 11.7-19.9). Being primiparity [AOR=5.798; 95% (1.572-21.377)], anemia during pregnancy [AOR=3.808; 95% (1.513-9.586)], pregnancy induced hypertension [AOR= 6.955; 95% (2.386- 20.275)], intake herbal medication during pregnancy [AOR=35.762; 95% (4.571-279.764)], drinking alcohol during pregnancy [AOR=8.111; 95% (2.359-27. 895)] were predictors of low birth weight.

**Conclusion:**

The proportion of low birth weight among newborns delivered at Wolaita Sodo University teaching and referral hospital was comparable with the global prevalence of low birth weight. Parity, anemia, alcohol, herbal medication, and pregnancy-induced hypertension were significantly associated with low birth weight.

## 1. Introduction

Low birth weight has a vital role in determining of newborns survival in a vulnerable condition. It tells fetal exposure to risk factors such as maternal unfavorable socioeconomic conditions, smoking habit, malnutrition, and diseases as well as lack of attention to prenatal care and delivery [1–3]. It also has a crucial role in estimating whether the newborn is at risk of death and disease during their neonatal period [4].

According to the world health organization (WHO) definition, babies with a birth weight of less than 2500g regardless of their gestational age are considered as low birth weight newborn [5]. Being low birth weight is associated with fetal and neonatal morbidity and mortality, impairment of growth and development and also chronic disease later in life [6].

Low birth weight (LBW) is still an important public health problem worldwide. Prevalence of Low birth weight across the globe accounts for 15.5%, which means that each year from 130 million annual births, 20 million is low birth weight [7]. Although there is variation in the number of low birth weight babies across regions, low and middle-income countries took a high figure particularly in most vulnerable populations [8]. The incidence of low birth weight in sub-Saharan countries estimates to be 13% to 15% [9]. According to Studies, in Ethiopia, there is a difference in the prevalence of low birth weight between geographical areas with a range from 8.4% to 28.3% [9–12].

Studies tested that various Socio-demographic, obstetrics and fetal factors are predictors of low birth weight. An institutional study in the northwest part of Ethiopia revealed being a preterm, absence of antenatal care, and malarial attack during pregnancy, anemia during pregnancy and lack of iron supplementation were the independent predisposing risk of low birth weight [13].

Though Wolaita Sodo University teaching and referral hospital is the only public referral hospital in the zone study on magnitude of low birth weight and its predictors were not available, studies in other parts of Ethiopia cannot represent a current study area due to a difference in socio-cultural, lifestyle and health-seeking behavior across the country.

Therefore, this study was aimed to assess the magnitude of low birth weight and its determinants irrespective gestational age and types of pregnancy in Wolaita Sodo teaching and referral hospital. Furthermore, the study will have input for program managers and policymakers in program designing, implementing and evaluating regarding neonatal mortality and also on improving childhood health care.

## 2. Methods and Materials

### 2.1. Study Design, Setting and Period

A hospital-based cross-sectional study was conducted in Wolaita Sodo University Teaching and Referral Hospital from December 1 to November 30. The hospital is found in Sodo town which is an administrative capital for the Wolaita zonal administration in South Ethiopia. The town is located at 380 km south from Addis Ababa. WSUTRH is the only public teaching and referral hospital in Wolaita Zone which provides a broad range of medical services to both in and outpatients of all age groups in its catchment area for about two million people. Based on a medical director report for 2017/18, around 3830 deliveries were attended in Wolaita Sdod university teaching and referral hospital in a single year.

### 2.2. Sampling Size and Sampling Procedure

The sample size was determined by using single population proportion formula with the assumption of 14.9% prevalence of low birth weight in North West Ethiopia [13], 95% confidence level and 4% margin of error. The sample size was calculated to be 304. Systematic random sampling with every other interval (K=2) was used to select study participants after review of a one-year delivery registration record to estimate the number of deliveries during the data collection time. If the selected mother-newborn pair does not meet the inclusion criteria or did not agree the next mother-newborn pair was taken. The first mother was selected by using the lottery method in the hospital.

### 2.3. Source and Study Population

The source populations were all neonates with their index mother who were delivered at Wolaita Sodo University teaching and referral hospital and the study populations were all neonates their index mother who were delivered at Wolaita Sodo University teaching and referral hospital during the data collection time.

### 2.4. Inclusion and Exclusion Criteria

All newborns delivered at Wolaita Sodo University teaching and referral hospital were eligible irrespective of their gestational age and parity except those delivered in other health institution or home but admitted into the hospital.

### 2.5. Data Collection Tool, Procedure and Quality Control

The data were collected through interviewing of a mother using a structured questionnaire, measurement of birth weight and review of medical records. The questionnaire was adapted from previous similar literature [13–15]. It had three parts; the first part was sociodemographic characteristics, the second part was obstetric and maternal characteristics, and the third part embraces neonatal related factors. Birth weight of naked newborn was measured within an hour of delivery before significant postnatal weight loss has occurred using balanced infant scale.

Data were collected by three BSc nurses who had experience of data collection and one-day training was given. The overall supervision was carried out by the principal investigators. A pre-test was conducted on 10% of the similar study population and appropriate modifications were made.

### 2.6. Data Processing and Analysis

The collected data were checked manually for completeness and entered into Epi-data version 3.1 and then exported to SPSS version 20. Univariate analysis was done using frequency and percentage. In bivariate analysis, candidate variables were recognized for multiple binary logistic regressions at a p-value of < 0.05. In multivariable logistic regression, odds ratios and 95% CI were computed and variables with p-value less than 0.05 were considered as significant.

### 2.7. Operational Definition

*Birth weight*: the first weight of the newborn which is measured before discharge

*Low birth weight*: neonate born with a birth weight of ≤2499g [5].

*Alcohol use*: a mother who took any unit of alcohol during the current pregnancy such as locally prepared alcoholic drinks (Tela, Teje, Areka), beer, wine or any alcoholic-liquors beverages

*Cigarette smoking*: smoking history of cigarette during the current pregnancy period even for once

*Herbal medicine*: herbs, herbal materials, herbal preparations and finished herbal products that contain as active ingredients parts of plants, or other plant materials, or combinations (36)

*Use of herbal/tradition medication*: a mother who took any unit of herbal medicine during the current pregnancy

*Anemia during pregnancy*: a mother whose hemoglobin (Hb) level is <11g/dl during the current pregnancy (37).

*Pregnancy-induced hypertension (PIH)*: A mother with high blood pressure (≥140/90mmHg) after 28 weeks of gestation of the current pregnancy and with or without proteinuria. Pregnancy-induced hypertension includes gestational hypertension, pre-eclampsia, and eclampsia (38).

### 2.8. Ethical Consideration

Ethical clearance was obtained from Wolaita Sodo University College of health science and medicine department of neonatal and pediatric nursing. An official letter of permission was written by the medical director of Wolaita Sodo University teaching and referral hospital. Mother of the participant neonate was informed about the purpose of study, anticipated benefit, full rights to refuse part or all of the study and as this study does not affect their future life. Written consent was obtained from her and confidentiality was assured during the data collection process. Other responsible authorities were also informed to get their support and cooperation for the study.

## 3. Result

### 3.1. Socio-Demographic Characteristics

In this study, a total of 304 mother-newborn pairs were included making100% response rate. The mean (±SD range) age of mothers was 25±5years ranging from 12 to 35 years and about three-fourth of the mothers' age range was between 20 and 34 years. About 261 (85.9%) and 189 (62.2%) mothers were Wolaita in ethnicity and Protestant in religion, respectively. About 56.3% of mothers were urban inhabitants. Of the total interviewed mothers, 175(66%) were housewives by occupation. The majority (46%) of mothers' educational status was secondary and above (Table 1).

### 3.2. Obstetric and Neonatal Related Factors

Maternal parity ranges from 1 to 7 with primiparity accounts for a larger proportion of 72.69% (221) compared to multiparity. 268 (88.2 %) pregnancies were a singleton and the rest 36 (11.8 %) multiple. Regarding antenatal care (ANC), 280 (92.1 %) respondents had ANC follow-up at least once during pregnancy of whom, and 143 (47%) mothers had ANC visit of four and above. Iron was taken by 213 (70.1%) mothers during the current pregnancy.

Forty-seven (15.5%) mothers had a history of abortion. Malaria by 70 (23%), pregnancy-induced hypertension by 33 (10.9%), diabetes by 13 (4.3%), premature rupture of the membrane by 15 (4.9%) and anemia by108 (35.5%) were documented as complications during the current pregnancy. Twelve (3.9%) mothers took traditional or herbal medication during this pregnancy. Alcohol and cigarette smoking were practiced by 23 (7.6%) and 24 (7.9%) mothers, respectively.

With regard to the mode of delivery, spontaneous vaginal delivery accounted for 156 (51.3%) mode of deliveries whereas instrumental and cesarean section were figured by 13 (4.3%) and 113 (44.4%) mothers, respectively. The duration of labor ranges from 1 to 34 hours, of which 110 (36.6%) mothers had experienced prolonged labor. One hundred fifty-four (50.7%) neonates were male in sex and the number of term and preterm newborns is 227 (74.7%) and 77 (24.3%), respectively.

### 3.3. The Proportion of Low Birth Weight

Of the total 304 study participants, 48 was low birth weight (LBW) which makes the proportion of LBW in Wolaita Sodo University teaching and referral hospital found to be 15.8%. The mean (±SD) birth weight of the newborns was 2971± 0.783gm ranging from 1600gm to 4800gm. Twenty-six (8.55%) low birth weight babies gestational age was 37 and above.

### 3.4. Bivariate Analyses of Independent Variables with Low Birth Weight

In binary logistic regression analyses, 11 variables showed significant association with low birth weight a p-value of <0.05 with 95% CI. Primiparity 2.381(1.022-5.549), antenatal follow up 3.708(1.518-9.055), maternal Co-morbidity during the current pregnancy, such as malaria 2.634(1.368-5.069), hypertension7.029 (3.235-15.273), and anemia2.508 (1.342-4.688).

Correspondingly, iron intake during current pregnancy 2.821(1.501-0.5.3), Premature rupture of membrane 7.029(3.235-15.273), history of abortion 2.105(1-4.43), and alcohol intake 14.091 (5.55-35.776) were significantly associated with low birth weight at COR (95%). Moreover, female newborns 0.363(0.186-0.709) and prematurity 3.092(1.628-5.872) had been associated with low birth weight (Table 2).

### 3.5. Factors Associated with Low Birth Weight

Multivariable binary logistic regression analysis was done by taking variables showing significant association on bivariate analysis at a p-value of ≤0.05 to adjust the possible confounding variables. Based on this, parity, anemia, hypertension, traditional medication, and alcohol were predictors of low birth weight at AOR (95%CI).

The finding of this study showed that the odds of low birth weight among primiparity mothers were five times more likely compared with those multipara mothers [AOR=5.798;95% (1.572- 21.377)]. Similarly, maternal co-morbidities during pregnancy, i.e., anemia and hypertension, had shown significant association with low birth weight. Low birth weight among anemic mothers was three times more likely compared with non-anemic mothers [AOR=3.808; 95 % (1.513- 9.586)]. Mothers who had a history of hypertension during current pregnancy were six times higher risk of getting low birth weight newborn compared to those no history of hypertension during current pregnancy[AOR=6.955;95%(2.386-20.275)].

Furthermore, intake of traditional or herbal medication and alcohol during pregnancy has a significant association with low birth weight. Mothers who took traditional medication during pregnancy were thirty-five times more likely to get low birth weight baby than mothers who did not take traditional medication [AOR=35.762; 95 %( 4.571-279.764)]. Newborn from a mother who drinks alcohol current pregnancy was eight times more likely be low birth weight compared to a mother who did not drink alcohol during current pregnancy [AOR=8.111;95%(2.359-27. 895)] (Table 3).

## 4. Discussion

World Health Organization (WHO) defines low birth weight as a birth weight of an infant 2499 gram or less irrespective of gestational age (5). The prevalence and risk factors of low birth vary across ethnicity, geography and economic status of countries. The study was aimed to assess the proportion and associated factors of low birth weight in order to reveal the burden of the problem and the possible local contributing factors. It was supposed that this study provided important information and created an overall image on the magnitude and associated factors of low birth weight at Wolaita Sodo University teaching and referral hospital.

The finding of this study revealed that the proportion of low birth weight in the study area was found to be 15.8% (95% CI 11.7-19.9). This finding was consistent with previous studies done in southern Ethiopia (17.88%), northwest Ethiopia (14.9%), Tigray region (14.6%), and Gondar town (17.4%) [13, 15–17]. However, this finding was higher than previous studies done

Axum (9.9%), Maichew (6.3%) and Jimma (11.01%) [10, 18], Nigeria (6.3%), United States and Canada [19–21]. The possible reason for this difference might be a variation in study time or mother's nutritional status and also the health professional's commitment to antenatal care service provision especially on dietary counseling during pregnancy.

On the other hand, this finding was lower than a finding in Debre Markose referral hospital Ethiopia (26.3%) [22]. This might be associated with antenatal care during pregnancy. A study in Debre Markose revealed that 11.9% (29) of mothers had not received any antenatal care (ANC) and those 77.3% (188) mothers had ANC visit one up to two times only, whereas in this study, 92.1% (280) of mothers had received ANC at least once during pregnancy of whom 47% of mothers had an ANC visit of four and above. World Health Organization (WHO) recommends antenatal care models with a minimum of four contacts by health professionals for better perinatal outcome [23].

Multivariate analyses of this study showed that the odds of low birth weight among primiparity mothers were 5.8 times more likely compared to multipara mothers. This study was in line with the study conducted in Nigeria and India [24, 25]. However, this finding was not in agreement with a study conducted in Tamilnadu which shows the prevalence of low birth weight was higher among multipara mothers [26].

Hypertensive mothers were at high risk of getting a low birth weight baby. The study showed that mothers who had a history of hypertension during current pregnancy were 6.9 times higher risk of getting low birth weight newborn compared to those mothers with no history of hypertension during the current pregnancy. This finding was supported by studies done in northwest Ethiopia and Nigeria [24, 27]. This could be due to the fact that hypertension during pregnancy is associated with under or poor perfusion of blood through placental and placenta is a vital organ which supplies blood and other essential nutrients to the fetus from its mother for normal growth and development [28, 29].

This study also assesses whether traditional (herbal) medications have an effect on birth weight of newborn since there is a cultural issue in the study area in which many more traditional malpractices are carried out. This study revealed that low birth weight among mothers who took traditional or herbal medications during the current pregnancy was 35.7 times more likely compared to mothers who did not take herbal medication. This might be due to the harmful effects of traditional medications. Using the traditional or herbal medicine during pregnancy might result in malnutrition, congenital anomaly, tumor or renal failure that had a direct effect on intrauterine growth restriction [30].

The finding revealed that the odds of low birth weight among mothers who had a habit of alcohol drink during the current pregnancy were 8.1 times more likely compared to those who did not drink alcohol during the current pregnancy. This finding was consistent with a study done Axum, Ethiopia [31]. This might be due to the fact that mothers who drink alcohol heavily during pregnancy might harm her fetus since alcohol can pass from the mother's blood into the fetus blood through the placenta and can damage the growth of the babies' cells, especially the brain and spinal cord cells. This further leads to low birth weight due to intrauterine growth retardation or restriction.

Moreover, this study showed that anemia was one risk factor of low birth weight. Low birth weight among anemic mothers was 3.8 times more likely compared with non-anemic mothers. This finding was supported by studies done in northwest Ethiopia and Sudan [13, 32]. This could be because anemia during pregnancy has an impact on the production of normal red blood cells that leads to low hemoglobin level. Low level of hemoglobin during pregnancy causes impairment on providing essential nutrients to the developing fetus which may compromise the normal growth. Furthermore, anemia is the complication of many systemic infections such as malaria, which further leads to intrauterine growth retardation [26, 33]

## 5. Conclusion

The proportion of low birth weight among newborns delivered at Wolaita Sodo University teaching and referral hospital was comparable with the global prevalence of low birth weight. Primiparity, anemia during the current pregnancy, traditional medication intake during pregnancy, pregnancy-induced hypertension and alcohol drink during pregnancy were predictors of low birth weight. Furthermore, this study recommends for all concerned body troublesome work on perinatal care to achieve sustainable development goal in Ethiopia since the low birth weight has an important role in neonatal mortality.

## Figures and Tables

**Table 1:** The socio-demographic characteristic distribution of mothers with their neonate at Wolaita Sodo University teaching and referral hospital, 2018.

Variable	Category	Frequency	Percentage (%)
Mothers age	<20	45	14.8
	20-34	235	77.3
	>35	24	7.9

Ethnicity	Wolaita	261	85.9
	Tigray	6	2
	Amhara	20	6.6
	Oromo	7	2.3
	Others	10	3.3

Religion	Protestant	189	62.2
	Orthodox	78	25.7
	Muslim	32	10.5
	Other	5	1.6

Marital status	Single	8	2.6
	Married	293	96.4
	Divorced	3	1.0

Residence	Urban	171	56.3
	Rural	133	43.7

Educational status	Unable to read and write	44	14.5
	Able to read and write	34	11.2
	Primary school	156	51.3
	Secondary and above	140	46

Occupation	Housewife	181	59.5
	Farmer	6	2.0
	Governmental employee	106	34.9
	Others	11	3.6

Mother dietary habit	Enjera	215	70.7
	Cereals	39	12.8
	Meat	13	4.3
	Starchy	32	10.5
	Fruit	5	1.6

**Table 2:** The association between low birth weight and independent variables among newborns delivered at Wolaita Sodo University teaching and referral hospital, Sothern Ethiopia, 2018.

Variable	Category	birth weight		COR	P-value
		Low	No low		
Parity	Primi	41	182	2.381(1.022-5.549)	0.044
	Multi	7	74	1	1
History abortion	Yes	12	35	2.105(1-4.43)	0.05
	No	36	221	1	1
ANC visit	Yes	39	241	1	1
	No	9	15	3.708(1.518-9.055)	0.004
Iron intake during pregnancy	Yes	24	189	1	1
	No	24	67	2.821(1.501-0.5.3)	0.001
Malaria during pregnancy	Yes	19	51	2.634(1.368-5.069)	0.004
	no	29	205	1	1
HTN	Yes	16	17	7.029(3.235-15.273)	<0.001
	No	32	239	1	1
PROM	YES	7	8	5.293(1.821-15.381)	0.002
	No	41	248	1	1
Anemia	Yes	26	82	2.508(1.342-4.688)	0.004
	No	22	174	1	1
Herbal medication	Yes	8	4	12.6(3.625-43.791)	<0.001
	No	40	252	1	1
Alcohol use during pregnancy	Yes	15	8	14.091(5.55-35.776)	<0.001
	No	33	248	1	1
Sex of neonate	Male	34	120	1	1
	Female	14	136	0.363(0.186-0.709)	0.003
Gestation(in weeks)	Preterm	22	55	3.092(1.628-5.872)	0.001
	Term	26	201	1	1

ANC=antenatal care; HTN=hypertension; PROM=premature rupture of membrane

**Table 3:** The association between low birth weight and different factors among newborns delivered at Wolaita Sodo University teaching and referral hospital, Sothern Ethiopia, 2018.

Variable	Category	birth weight		AOR	P-value
		Low	No low		
Parity	Primi	41	182	5.798(1.572-21.377)	0.008
	Multi	7	74	1	1
History abortion	Yes	12	35	1.189(0.399-3.541)	0.755
	No	36	221	1	1
ANC visit	Yes	39	241	1	1
	No	9	15	0.289(0.051-1.632)	0.16
Iron intake during pregnancy	Yes	24	189	1	1
	No	24	67	1.032(0.396-2.689)	0.948
Malaria during pregnancy	Yes	19	51	1.547(0.59-4.053)	0.375
	no	29	205	1	1
HTN	Yes	16	17	6.955(2.386-20.275)	<0.001
	No	32	239	1	1
PROM	YES	7	8	2.942(0.596-14.517)	0.185
	No	41	248	1	1
Anemia	Yes	26	82	3.808(1.513-9.586)	0.005
	No	22	174	1	1
Herbal medication	Yes	8	4	35.762(4.571-279.764)	0.001
	No	40	252	1	1
Alcohol use during pregnancy	Yes	15	8	8.111(2.359-27. 895)	0.001
	No	33	248	1	1
Sex of neonate	Male	34	120	1	1
	Female	14	136	0.574(0.249-1.32)	0.191
Gestation(in weeks)	Preterm	22	55	1.983(0.787-4.996)	0.147
	Term	26	201	1	1

ANC=antenatal care; HTN=hypertension; PROM=premature rupture of membrane

## Data Availability

The data used to support the findings of this study are available from the corresponding author upon request.
